# Employee and Family Assistance Video Counseling Program: A Post Launch Retrospective Comparison With In-Person Counseling Outcomes

**DOI:** 10.2196/med20.3125

**Published:** 2014-04-24

**Authors:** Barbara Veder, Stan Pope, Michèle Mani, Kelly Beaudoin, Janice Ritchie

**Affiliations:** ^1^Shepell·fgiOttawa, ONCanada; ^2^Shepell·fgiToronto, ONCanada

**Keywords:** EAP, EFAP, counseling, video counseling, technology, mental health, online counseling, therapy, online therapy

## Abstract

**Background:**

Access to technologically mediated information and services under the umbrella of mental and physical health has become increasingly available to clients via Internet modalities, according to a recent study. In May 2010, video counseling was added to the counseling services offered through the Employee and Family Assistance Program at Shepell·fgi as a pilot project with a full operational launch in September 2011.

**Objective:**

The objective of this study was to conduct a retrospective post launch examination of the video counseling service through an analysis of the reported clinical outcomes of video and in-person counseling modalities.

**Methods:**

A chronological sample of 68 video counseling (VC) cases and 68 in-person (IP) cases were collected from a pool of client clinical files closed in 2012. To minimize the variables impacting the study and maintain as much clinical continuity as possible, the IP and the VC clients must have attended clinical sessions with any one of six counselors who provided both the VC and the IP services. The study compared the two counseling modalities along the following data points (see glossary of terms): (1) client demographic profiles (eg, age, gender, whether the sessions involved individuals or conjoint sessions with couples or families, etc), (2) presenting issue, (3) average session hours, (4) client rating of session helpfulness, (5) rates of goal completion, (6) client withdrawal rates, (7) no show and late cancellation rates, and (8) pre/post client self-assessment. Specific to VC, we examined client geographic location.

**Results:**

Data analysis demonstrates that the VC and the IP showed a similar representation of presenting issues with nearly identical outcomes for client ratings of session helpfulness, rates of goal completion, pre/post client self-assessment, average session duration, and client geographic location. There were no statistically significant differences in the rates of withdrawal from counseling, no shows, and late cancellations between the VC and the IP counseling. The statistical analysis of the data was done on SPSS statistical software using 2-sample and pairwise comparison *t* tests at a 95% level of significance.

**Conclusions:**

Based on the study, VC and IP show similar outcomes in terms of client rating of session and goal attainment.

## Introduction

### Web-Based Mental Health Services

With continual technology advancements, and greater access, Web-based mental health services are increasingly being offered to a range of client populations [1-4]. Furthermore, there is growing interest in these advances with regard to improving client/patient accessibility to services (including assessment and treatment) [5]. Web-based self-help tools are also expected to increase in number and variety [3].

The importance of adapting Employee and Family Assistance Program (EFAP) counseling to technological innovations, to better serve client needs with new tools and services, is supported by meta-analysis of the effectiveness of Web-based therapeutic interventions [6].

Shepell·fgi offers a wide range of services to its organizational clients, their employees, and families. In response to changes in technology and client needs, Shepell·fgi developed several Web-based counseling platforms, clinical options, and self-directed tools for clients.

The asynchronous e-counseling service was introduced in 2000. Self-directed Web-based tools (such as stress reduction) are also available to individual and organizational clients. The MyEAP app was launched in May 2011. The same year, First Chat—a 24/7 synchronous live “chat” option for clients who want immediate clinical and/or intake support—was designed and offered to clients.

To further expand Web-based counseling options, and to offer a Web-based synchronous counseling modality for clients living in rural or remote areas, Shepell·fgi developed and launched their video counseling program as a pilot project in May 2011, offering video counseling (VC) to a limited number of organizational clients (and their employees). The EFAP VC program was subsequently launched as a core clinical service and made available to a broad range of eligible EFAP clients in September 2011, and, in 2012, 722 cases were opened.

Only modest technical abilities are required by clients in order to successfully participate in VC, making it accessible to most. The client and counselor communicate using a webcam, landline, and encrypted custom Internet software. Both parties can see and hear each other, and they can also share and create documents in real time. Clients can use their personal computers at home for this counseling.

Much anecdotal evidence suggests that Shepell·fgi VC EFAP clients find VC clinically helpful and a convenient and beneficial service. Completed satisfaction surveys, providing quantitative and qualitative feedback, indicate that clients are satisfied with the service received. To date, no formal or informal service complaints or client requests to change counseling modalities have been received.

Some of the advantages cited by clients are- time factors, reduced travel, and increased convenience with regard to child care and family responsibilities.

The purpose of this paper is twofold: (1) to examine specific data post VC launch, and determine if anecdotal evidence is supported by various outcome measures (eg, client session effectiveness rating, pre/post self-assessment, client goal attainment ratings, case withdrawal rates, and average session hours); and (2) to compare these clinical outcome factors with the same EFAP in-person counseling (IP) client outcome measures.

### Data Collection

The data were collected from closed clinical files of VC (n=68) and IP (n=68) clients. The clients from both samples initiated counseling sessions within the same time range, VC from July 2011 to September 2012, and IP from June 2011 to October 2012. The IP and VC client demographic information was also examined and compared. The clinical files used for this study were drawn from the closed clinical records of six counselors who provided both VC and IP services.

With regard to client demographic information, the authors expected more women than men to be represented in both the IP and VC samples. Shepell·fgi’s annual statistical analysis shows that more women access clinical services across multiple modalities including First Chat, self-directed/self-help resources, Web-based self-help resources, traditional IP, tele-counseling, and e-counseling.

Informed by previous research studies (discussed below), as well as EFAP VC counselor and client feedback, we hypothesized the following, the VC clinical outcomes (as defined in this paper) would be similar to the IP clinical outcomes; clients would report high satisfaction with the VC sessions; and no marked differences between VC and IP would be observed on the clinical measures examined in this study.

For the purposes of this paper, although VC is the term that is most often used when discussing the EFAP service, other terms (eg, tele-mental health, TMH; telehealth, TH; and teleconferencing) will also be referenced.

### Current Research

Current research findings suggest that clients using VC report high levels of satisfaction, with similar satisfaction and clinical outcomes to clients accessing IP. Several extensive research literature reviews support this finding [2,3,7]. Some of the issues compared in the reviewed studies include clinical effectiveness, client satisfaction, modality equivalency, and/or efficacy. The reviewed research represents different mental health providers and professions, using a variety of clinical approaches (eg, cognitive behavioral therapy, CBT; psychiatric assessment and follow-up; different clinical models; etc). They also include a wide range of client populations, ages, and various clinical/mental health issues.

Also relevant is a systematic literature review [5] that focused on the therapeutic interventions delivered by videoconferencing for long-term and chronic mental and physical health issues. The reviewers identified certain methodology limitations in some of the studies, but also found high quality randomized controlled trials to examine. As an outcome of the review, they reported that the videoconferencing interventions produced similar outcomes, patient satisfaction, and treatment results in regards to patients who received in-person interventions. No recent research in their scope of review suggested videoconferencing and face-to-face interventions were dissimilar [8].

In partial contrast, a 2010 systematic review, based on 11 articles published pre 2009 with defined study criteria, reported “there is insufficient scientific evidence regarding the effectiveness of telepsychiatry in the management of mental illness” [9]. At the same time, the authors reported that their findings support videoconferencing as “feasible and effective”, and noted the high levels of satisfaction reported by patients. Furthermore, along with recommendations of further research, they highlighted the key role telepsychiatry can play going forward in providing high quality care to patients [9].

In reviewing the literature and developing their research, O’Reilly et al [10] brought specific attention to the importance of not assuming “equivalence” when studies show a lack of statistical differences in outcomes. In their randomized controlled equivalence trial comparing telepsychiatry with face-to-face sessions, they found both modality subjects shared equivalent clinical outcomes and reported similar satisfaction rates. At the same time, they remarked that the equivalence outcomes found in their study might not be replicable to other mental health services, such as psychotherapy [10]. Many researchers and literature reviewers noted similar limitations with regards to available research, and noted similar considerations and implications for future research. Mainly discussed was the need for larger sample groups, replicable interventions, study design limitations, and the lack of randomized clinical trials. The importance of developing a standard evaluation model and methodologies was also highlighted [11].

At the same time, the current literature reviews and analysis cited above suggest that on the whole, there were similar and comparable clinical outcomes and client satisfaction between clients/patients who received VC and IP.

More recent literature reviews and individual studies also seem to support the finding of similar patient outcomes and satisfaction levels between VC and IP clients.

Steel et al [8] conducted a substantive review of video interventions for the treatment of long-term and chronic mental and physical health. Their review included a number of high quality randomized controlled trial studies, and summarized that patients receiving videoconferencing interventions (for a variety of physical and mental conditions) demonstrated similar treatment outcomes and satisfaction levels to IP [8].

A compelling study [5] examined and compared TH and in-person treatment outcomes of US veterans diagnosed with post traumatic stress disorder (PTSD). There were 12 exposure therapy sessions that were delivered to the veteran patients by means of TH or in-person therapy. The researchers reported effective outcomes for the veterans from TH exposure therapy. When comparing the IP and TH samples, they also found exposure therapy via IP was more effective than when delivered via TH. The authors speak directly to this result and propose possible reasons, including an above average IP effect size (when compared to other published averages) observed for in-person exposure therapy and the lack of randomization. At the same time, they found and concluded “brief TH exposure therapy was effective in treating the symptoms of PTSD, depression, anxiety, and general impairment in veterans with PTSD,” and no significant differences in outcome effects were found across demographic groups [5].

Of considerable interest is the groundbreaking 2012 study representing the largest scale assessment of TMH [12]. This study assessed clinical outcomes of 98,609 US Department of Veteran Affairs (VA) patients over four years (2006-2010). TMH services were provided to veterans at community-based outpatient clinics by a wide range of mental health practitioners (such as psychiatrists, psychologists, social workers, and registered nurses). The findings included that patients receiving TMH services not only had fewer days of hospitalization, but an average of 25% fewer hospitalizations [12]. Although there was no control group, they were able to identify “the overall VA population of mental health patients did not demonstrate similar decreases during this period.” This includes VA patients receiving other forms of mental health services.

Although therapeutic alliance is outside the scope of this specific research, it is nevertheless a key clinical component and process variable. The authors explored research on therapeutic alliance, as it is relevant to VC/videoconferencing. Richardson et al [2] examined several studies that looked at alliance. These include Ruskin et al [13], who reported a robust development of therapeutic alliance; Cluver et al [14], who found patients rated the quality of alliance similarly in both in-person and videoconferencing services; and Grady and Melcer [15], who found no significant differences in therapeutic alliance ratings when analyzing in-person and TMH services delivered to military personnel.

The Web-based counseling literature review performed by Mallen et al [3] also discussed studies that found adult clients reported similar therapeutic alliance between videoconferencing and in-person services. Steel et al [8] discussed the possibility of developing a good therapeutic alliance through videoconferencing. Finally, Rees and Stone [16] summarized their findings of therapeutic alliance in videoconferencing versus in-person psychotherapy as, “the current literature indicates that therapeutic alliance is not compromised when videoconferencing is used.” Interestingly, their research (a sample of 30 psychologists) found that psychologists conducting VC sessions rated therapeutic alliance lower than psychologists in face-to-face sessions. Other research also supported this, and found that psychologists who used this modality rated therapeutic alliance lower than their clients. Rees and Stone discussed possible reasons why psychologists might hold these negative beliefs, and proposed approaches to reduce them.

### Accessibility and Underserved Populations

Improving accessibility to populations living in remote and underserved areas was a key factor in the EFAP's decision to develop VC services. Other researchers and practitioners investigating the potentials of VC echo these considerations.

Many articles discuss the intrinsic possibilities and benefits of mental health services via VC to different clinical populations. Identified potential populations who would benefit from VC include people living in remote areas, underserved populations (including multicultural minorities), marginalized populations, and differently-abled individuals [2,3,16]. The benefit of expanding TH services to better serve clients in need has also been highlighted [5].

In their review of current research, Steel et al [8] found a number of literature reviews that reported that the use of teleconferencing led to increased service access in the United Kingdom.

In particular, Myers and Turvey [17] noted how the use of technology could assist access to specialized services/providers. Moreover, in the article, “Use of standard webcam and internet equipment for telepsychiatry and treatment of depression among underserved Hispanics," Moreno et al [18] describe strong benefits from using lower cost, nonsophisticated, teleconferencing tools (via the Internet), making this modality accessible to many populations.

VC was made available to EFAP clients in urban, rural, and remote locations. This study may also provide useful information with regard to client populations with nonpsychiatric presenting issues who also may benefit from VC. Local clinics, universities, health centers, other EFAPs, and even private practitioners might treat a similar client base. Considering the range of client background and presenting issues, this research can add to the current literature for this promising area of study.

VC provides client access possibilities that are related to other factors as well. Some clients who might be disinclined to attend more traditional IP may view VC/TMH as a viable alternative. Some clients may be hesitant to access face-to-face services for many reasons, including perceived stigma [16]. Likewise, convenience and availability factors can play an important role in modality preference for some clients [3]. Technology services are also a viable option for the clients who do not like certain features of in-person support [19]. Of interest are the possible outcome effects of Web-based counseling, such as clients feeling less dependent on their counselor, and potentially experiencing “greater equality in the sessions” [3].

## Methods

### The Client Sample

The client sample was selected retrospectively, from closed clinical files, which do not contain identifiable information. At the onset of the first counseling session, clients are informed of and consent to the Statement of Understanding, which indicates nonidentifiable data may be used for research purposes.

For the purposes of this study, Shepell·fgi staff collected and examined a sample of 68 VC cases, opened in a 14 month period between July 2011 and September 2012, and 68 IP cases, opened between June 2011 and October 2012, for comparison. The compared cases were collected from a pool of both VC and IP cases closed in 2012. As clinical files were chosen chronologically, the clients represented a wide range of ages, geographic locations across Canada, and presenting issues. The clients were predominantly English speaking; however, there were French-speaking clients in both counseling modalities.

There were 6 EFAP counselors from Ontario, Quebec, British Columbia, and the Northwest Territories who provided both IP and VC counseling that were identified. The counselors were all Masters’ level mental health professionals from across Canada with different professional backgrounds, including social work, psychology, and counseling, with five or more years of experience. All were trained in providing short-term counseling using CBT and solution focused skills. They also brought their individual experiences, clinical competencies, and aptitudes to their work. 

Starting with files closed in January 2012, a similar number of VC and IP cases were pulled, in chronological order, for each counselor.

The following files were discounted from the selection of the complete list of cases assigned to these counselors: (1) files where the client did not materialize for the first or subsequent appointments, and consequently the file was closed; and (2) files that were recorded as closed, but clinical documentation had not yet been submitted.

Once those files were filtered out, the study sample of 68 VC and 68 IP files were pulled from this chronological list.

Statistical analysis of the data was done on SPSS statistical software using 2-sample and pairwise comparison *t* tests at a 95% level of significance.

The study compared the two modalities along the following data points (see Multimedia Appendix 1 for a glossary of terms): (1) client demographic profiles (eg, age, gender, whether the sessions involved individuals or conjoint sessions with couples or families, etc), (2) presenting issues, (3) average session hours, (4) client rating of session helpfulness, (5) rates of goal completion, (6) client withdrawal rates, (7) no shows/late cancellations, and (8) pre/post client self-assessment. Also, specific to VC, we examined client geographic location.

Location differences were examined, as improving client accessibility to clinical service was a key rationale in the development of VC.

As far as the differences between the two samples, all IP sample cases comprised clients residing in an urban setting who could access IP with less than 30 minutes of travel.

In comparison, of the 68 sample VC cases, 69% (n=47) were easy access (within 30 minutes of an IP EFAP counselor), 25% (n=17) were classified as moderate access (within an hour), and 6% (n=4) were limited/no access (more than an hour away.)

### Inclusion/Exclusion Criteria

Inclusions and exclusions of clients for the sample were based on the referral process for the modalities. The clients, upon contacting the EFAP to request counseling support, were assigned to either the IP or VC modality based on two factors: (1) they specifically request one of the modalities; or (2) intake recommends IP or VC after assessing the client’s preferences and needs. The factors that influence the recommendations are explained in a multimedia file (see Multimedia Appendix 2). The decision to accept the referral recommendation for either service modality is made by the client.

The clients are assigned to the VC modality if: (1) their presenting issues are not high risk (eg, if a client reports that they are not at risk of harming themself or others; or has low addiction issues), (2) they meet the technological requirements as shown in a multimedia file (see Multimedia Appendix 3), and (3) they are over 18 years old.

The clients assigned to the IP modality do not need to meet the same exclusion criteria, as do those assigned to VC. However, for this study, the IP clients under the age of 18, and those presenting with high risk issues were excluded.

Once the sample files were identified, the research team obtained the clinical files from storage. Each file was then reviewed, and the data points used in the study were charted.  

## Results

### Demographics of the Sample

Table 1 shows the breakdown of various demographics of the sample.

Both VC and IP had similar demographics in terms of client age and gender. Women accessed EFAP counseling more than men, and individuals accessed this counseling more often than couples/families.

All of the IP clients resided in regions with easy access to IP services. Of the VC clients, 69% (47/68) resided in regions with easy access to IP services, 27% (18/68) in hard to service locations, and 4% (3/68) resided in the hardest to serve locations.

### The Presenting Issues

The presenting issues were divided into four main areas: (1) addiction, (2) couple/family relations, (3) personal/emotional adjustment, and (4) workplace issues. Both the VC and IP sample cases showed a similar distribution across these issues.

The cases ranged from one to seven hours. The average case duration was 3.91 hours for the VC and 4.07 hours for the IP (Table 2).

There was no statistical difference in the average rating of session helpfulness at a 95% level of significance between the VC and IP modalities. Not all sessions received a client rating. For the 68 VC cases, 117 out of 173 sessions received a client rating, and the average client rating for these sessions was 8.5 out of 10.0. For the 68 IP cases, 131 out of 184 sessions received a client rating, and the average was 8.6 out of 10.0 (Tables 2 and 3).

The differences in goal completion were also not statistically significant at a 95% confidence in rates of goal completion. VC cases had a goal completion percentage of 84% (57/68), and IP cases a goal completion percentage of 71% (48/68) (Table 4).

The rate of client withdrawal from counseling showed no significant difference at a 95% level of significance. The VC withdrawal rate was 16% (11/68), and the IP withdrawal rate was 28% (19/68). Modality redirects, (clients changing counseling modalities; eg, from VC to IP), only occurred once in the VC sample cases (Table 5).

There was a marked difference in the category of client no shows and/or late cancellations, in that the rate of client no shows and/or late cancellations was 11.6% (20/173) for the VC cases, and 19.0% (35/184) for the IP cases (Table 6). This difference was found to be statistically significant at a 95% level of significance.

Table 7 shows that pre/post assessment of client ratings of health and mental health showed similar results. The IP cases demonstrated a net improvement of 10% (3.14/5 to 3.45/5) on the health question, and a net increase of 22% (2.64/5 to 3.21/5) for the mental health rating. This difference was not statistically significant.

For the VC cases, there was a net improvement of 11% (3.03/5 to 3.36/5) on the health rating. There was also a net improvement of 11% (2.89/5 to 3.21/5) for the mental health rating.

**Table 1:** Summary of the characteristics of the study sample.

Characteristics		VC (n=68)	IP (n=68)
**Demographics**			
	Age	39	38
	Female %, (n)	66 (45)	58 (39)
	Male %, (n)	34 (23)	42 (29)
**Client location %, (n)**			
	Easy access	69 (47)	100 (68)
	Moderate access	27 (18)	0 (0)
	Limited/no access	4 (3)	0 (0)
**Type %, (n)**			
	Individual	78 (53)	88 (60)
	Conjoint	22 (15)	12 (8)
**Presenting issue %, (n)**			
	Addiction	2 (1)	6 (4)
	Couple/family	47 (32)	31 (21)
	Personal/emotional	44 (30)	59 (40)
	Work related	7 (5)	4 (3)

**Table 2:** Summary of case/session data.

Dimensions	VC	IP
Average case hours, range = 1-7	3.91	4.07
Client session rating, average out of 10.0	8.5	8.6
Goals attained/goals partially attained %, (n)	91 (52/57)	96 (48/50)
Withdrawal rate %, (n)	16 (11/68)	28 (19/68)
No show/late cancellation rate %, (n)	12 (20/173)	19 (35/184)

**Table 3:** Comparison of session helpfulness ratings.

Session helpfulness	n	Mean	SD	Standard error mean
IP	131	8.7328	1.23926	.10827
VC	117	8.5855	1.20038	.11098

**Table 4:** Comparison of goal completion.

Goal completion	n	Mean	SD	Standard error mean
IP	50	.9000	.24744	.03499
VC	57	.8684	.30657	.04061

**Table 5:** Comparison of withdrawal rates.

Withdrawal rates	n	Mean	SD	Standard error mean
IP	68	.28	.452	.055
VC	68	.16	.371	.045

**Table 6:** Comparison of no show and late cancellation rates.

No shows/late cancellations	Number of events X	Total number of samples N=357	Proportion of no shows *P*
IP	35	183	.19125
VC	20	174	.11494

**Table 7:** Summary of pre/post questionnaire results.

Cases		VC (n=30)	IP (n=35)
**With improved health rating**			
	Pre health rating average out of 5.00, (n)	3.03 (8)	3.14 (11)
	Post health rating average out of 5.00, (n)	3.36 (8)	3.45 (11)
**With improved mental health rating**			
	Pre mental health rating average out of 5.00, (n)	2.89 (9)	2.64 (16)
	Post mental health rating average out of 5.00, (n)	3.21 (9)	3.21 (16)

## Discussion

### Principal Findings

This study reviews the VC program one year post launch. It examines the data points to determine if the specific outcome measures support the anecdotal VC feedback that was received. It compared the VC client clinical outcome measures with those of the IP clients. The decision to conduct this preliminary research was made in order to evaluate the EFAP’s VC clinical service, to gain a greater understanding of the client population, and to contribute to the current VC literature. The past decade has seen a significant technological evolution; making the use of VC/TMH/TH increasingly feasible and available to different providers and populations. The expansion of this modality, the possibilities for clients, and the growing breadth of research are exciting developments.

The clinical management became aware of the VC positive feedback from the video counselors during clinical supervision, and from the client satisfaction surveys they received. The clinical services are monitored for positive feedback and/or clinical indicators, as well as formal/informal negative client feedback or complaints. VC received neither informal/formal negative client feedback nor complaints.

Shepell·fgi recognized the opportunity to compare VC with IP, to research the clinical outcomes from the counselors who delivered both VC and IP services to the EFAP clients. Furthermore, both of these modalities use the same case management and case files.

It was determined that the VC and the IP clients would be compared according to the dimensions noted above. A primary measure of comparison was based on direct client session ratings. While not all of the sessions received a rating, the majority of them did. By this measure, there was no statistical difference in how clients rated the usefulness of the VC sessions as compared with the IP ones. Both of them received high client ratings, with an average of 8.5/10 for VC, and 8.6/10 for IP.

Goal attainment (attained, partly attained, or not attained) is a more subjective rating. However, the rates of VC (91%, 52/57) for goal attainment were on par with IP (96%, 48/50), and the difference was not statistically significant.

It appeared that there were differences in the area of withdrawal from counseling and clients not showing for scheduled appointments. The VC clients showed a lower rate of withdrawals and no shows. The withdrawal rate from VC was measured at 16% (11/68), and the IP withdrawal rate was measured at 28% (19/68). There was also a difference in the no show rate, 11.6% (20/173) for VC and 19.0% (35/184) for IP. The data analysis indicated that there was no statistical significance to the withdrawal rates between the modalities; however, there was evidence to support that the no show/late cancellations rates are statistically lower for VC cases than for IP cases.

Demographically, the two samples were similar in terms of age, with an average age of 39 for VC and 38 for IP. There were a slightly higher percentage of female users of VC (66%, 45/68; vs 57%, 39/68). This finding is congruent with EFAP gender findings as, averaged across modalities, women represented 70% of the 2012 EFAP cases.

As expected, the geographical distribution of the two samples was different. The clients in the IP group were in regions with easy access to IP services, and it was expected that the VC clients would predominantly be from hard to serve regions. It is interesting to note that 69% (47/68) of the VC services were provided to clients located in regions with easy to access IP. This indicates that clients chose VC even when IP was readily available. Further research outside the scope of this study is needed to clarify these findings.

The pre/post questionnaire results showed some differences between VC and IP. The sample size for this measure was reduced, as only questionnaires completed both pre and post counseling were used. For VC, 30 of 68 questionnaires met the criteria, and for IP, 35 of 68 were fully completed.

In terms of the health response, both IP and VC showed improvement in the post counseling health measure. There were 8 out of 30 VC and 11 out of 35 IP cases that noted improvement in health. The average health score increased by 11% (3.03/5 to 3.36) for VC and 10% (3.14/5 to 3.45/5) for IP. These results were acceptable given that most of the clients do not access EFAP counseling to manage physical health issues.

For the mental health response, both IP and VC also showed modest improvement in this post counseling health measure. Only 9 out of 30 (30%) VC cases and 16 out of 35 IP cases (46%) noted improvement in mental health. The average mental health score increased by 11% (2.89/5 to 3.21/5) for VC clients and 22% (2.64/5 to 3.21/5) for IP clients. A possible reason for these rates is that not all clients access EFAP services for mental health concerns (eg, workplace issues, marital issues, family concerns, etc). If the clients did not rate mental health as a concern at the case outset, improvement in this area is moot.

Within the group of cases with completed pre/post questionnaires, it was found that, of the VC sample (30 cases), 8 individual cases rated their precounseling mental health as only poor or fair and, of these cases, 6 (75%) reported improvement. Similarly, of the 35 completed IP pre/post questionnaires, 14 individuals rated their mental health as only fair or poor in the preevaluation, and, of these, 11 individuals (79%) reported improvement. While this subset makes for a small sample, these findings are consistent with the hypothesis that VC would show similar clinical outcomes to IP.

An area of surprise was the difference in the rate of conjoint counseling for the two modalities. Initially, a higher rate of conjoint counseling for IP versus VC was expected. However, the research did not support this. To the contrary, 22% (15/68) of the VC cases were for conjoint counseling, while only 12% (8/68) of the IP cases were conjoint. It can be hypothesized that the ease of access to VC in terms of location and times makes it easier for conjoint counseling. The IP clients are constrained in terms of travel time, and they must operate in the same time zone as the counselors, thus restricting the availability of evening appointments. For the VC clients who live in an eastern time zone, there is greater availability for evening appointments with western VC counselors (eg, a client from Toronto may have a 9:00 p.m. Eastern Standard Time appointment with a Vancouver counselor who is working at 6:00 p.m. Pacific Standard Time).

In addition, as VC typically takes place in the client’s home, barriers are reduced with regard to coordinating conjoint clients’ schedules and child care arrangements.

There was virtually no difference in the average number of sessions (3.91 sessions for VC vs 4.07 for IP, where each case ranges from 1-7 sessions).

The presenting issues referred to the types of problems that clients presented at intake. As the relatively high rate of conjoint counseling for the VC sample would indicate, couple/family issues were greater in the VC sample (47%, 32/68) than for the IP sample (31%, 21/68). The work related and addiction categories only accounted for six cases in the VC group, and seven cases in the IP sample.

The above highlights the finding of similar clinical outcomes between VC and IP adult EFAP clients. It also highlights the differences with regards to higher IP pre/post mental health results, and the higher rate of couples/families presenting issues in VC.

This appears to support the existing literature that suggests similar clinical outcomes between VC and IP, and/or a high rate of VC client satisfaction.

### Other Research: Current Areas of Exploration

The research reviewed previously in this paper reflects the different methodologies used, the client/patient populations, the presenting issues/diagnoses, the mental health professions, and the clinical models (eg, CBT; psychiatric assessment and follow-up).

There are studies that used manualized treatment provided by mental health practitioners from different theoretical orientations (eg, CBT; exposure therapy), and research where one mental health orientation was represented using various interventions (eg, psychiatrists offering service to rural clients) [5,10]. 

Some methodologies included mental health providers who serviced both VC and IP clients; others provided service to only VC or IP clients; and some used single and multi-modality providers [5,10]. The research featured some clients who were mostly “seen” (via TMH) in offices or clinics, as well as other clients attending sessions from their homes [5,8,12,18]. The studies tested for TMH equivalence or similar outcomes to face-to-face interventions, effectiveness, and therapeutic alliance, as well client satisfaction ratings [6,16].

Prior studies’ methodology included using randomly assigned client samples and nonrandomly assigned VTH samples [5,10,18]. The study models included psychiatric evaluation and brief follow-up, as well as mental health professionals providing clinical therapy over multiple sessions [5,18]. The studies included data when client post scales were not completed by the total sample, and when a percentage of clients did not complete the actual treatment. 

Some studies focused on one particular cultural group and presenting issue or diagnosis; while others studied patients with a general diagnosis that could encompass multiple issues such as chronic conditions, depression, anxiety, and general impairment related to PTSD [5,8,18]. Taken together, the studies encompass a rich variety of clients, issues, mental health professionals, and methodological approaches.

In their exposure therapy study for veterans with PTSD, Gros et al [5] noted how using a “standard clinical practice, rather than a highly controlled research setting, (emphasized) the potential for widespread dissemination and implementation of TH treatments.” This paper’s authors agree that more widespread implementation of video intervention could be beneficial. 

### Study Limitations

The authors are aware of several limitations of the “EFAP Video Counseling: A Post Launch Retrospective and Comparison With In-Person Counseling Outcomes” study.

Although the research used a larger sample size than many studies, it remains modest with regard to offering statistically significant data. Using larger sample sizes in future studies would be consistent with other VC research being currently conducted, provide more insight, and offer interesting outcomes.

Lack of a control group and nonrandom sample selection are other limitations. Due to the nature of the EFAP service, this is unlikely to change in future research. The EFAP offers services to its clients relating to stated client preference, described lifestyle, and/or recommendations based on the client’s stated issue. A control group or random modality assignment would not reflect the best possible clinical service for a client, which remains a priority.

Another limitation in the study was using subjective rating tools. The pre /post assessment and session rating helpfulness scale are completed by the client in the presence of the counselor (and the latter administered only when deemed clinically appropriate by the counselor), which may affect the client’s response. At the same time, it is important to note that counselors are trained to present the scales as a helpful tool for the client and the counselor, an indicator to see if they are moving in the preferred direction, or if a different approach would be helpful. The clients are encouraged to actively cocreate session direction and focus.

The client sample includes self-referred people from various sociocultural and economic backgrounds who live across Canada in isolated, rural, and urban communities, English and French speakers, individuals, and couples who present with a wide range of concerns and clinical goals. Although the diverse population and clinical issues may be perceived as a study limitation (it does not compare the same populations), it can also be seen as a study strength. It reflects a rich diversity within the EFAP client population and communities across the country.

### Future Research

This study provides useful information for exploring other client populations with nonpsychiatric presenting issues who may also benefit from VC. Local clinics, universities, health centers, other EFAPs, and even private practitioners might provide VC as an addition to IP services. Future research using larger sample sizes would be consistent with other VC research currently being conducted, provide more insight, and offer interesting outcomes. Furthermore, client populations with more specific presenting issues (eg, clients identified with depression or anxiety) can provide additional data for this promising area of study.

While most of the research studies cited in this paper reflect video and Web-based services provided to individuals, the future study of VC with couples and families to ascertain its helpfulness (as compared to IP) could prove an interesting area of inquiry.

While recognizing the limitations in methodology, the findings remain interesting and suggest future research possibilities. The technology used is accessible to many Canadians at low cost, and enables clients to participate in counseling sessions in their own home.

### Conclusions

The EFAP, through its capacity to offer multi-modal clinical services to thousands of clients a year across client demographics, locales, and presenting issues, is in a unique position to add to the current literature in this area of study. For many working people in Canada, the EFAP is the easiest and most effective way to access timely, confidential, and no-cost counseling.

Moreover, as the majority of the client base accesses the EFAP with nonpsychiatric presenting issues, the EFAP clients are an important and underrepresented population in the current research. Their presenting issues include relationships, grief, depression, and stress. These areas of concern correspond with many other clients who seek short-term counseling.

The findings of this study demonstrate similar VC and IP clinical outcomes as demonstrated by client attendance, rate of session helpfulness, pre/post self-assessment, and rates of goal completion.

## Multimedia Appendix 1

Glossary of terms.

## Multimedia Appendix 2

Clinical intake screening process.

## Multimedia Appendix 3

Technological requirements for video counseling.

## Abbreviations

CBT: Cognitive behavioral therapy
EFAP: employee and family assistance program
IP: in-person counseling
PTSD: post traumatic stress disorder
TH: Telehealth
TMH: tele-mental health
VA: Veteran Affairs
VC: video counseling
