# Supplementary material for: Employee and Family Assistance Video Counseling Program: A Post Launch Retrospective Comparison With In-Person Counseling Outcomes
Source: Med 2 0. 2014 Apr 24;3(1):e3. doi: 10.2196/med20.3125 (PMC4084764; doi:10.2196/med20.3125)
Supplement: Supplementary file 1 [file med20_v3i1e3_app1.pdf]

Multimedia Appendix 1: Glossary of terms used in this article

EFAP	Services purchased by the employer as part of the employee's benefits package include integrated health and productivity solutions that address the mental, physical and social health issues affecting the workplace, the employees and their families.
Video counselling	Synchronous, confidential counselling service where client and counsellor communicate using a webcam, landline, and encrypted custom internet software. Both parties are able to see and hear each other and can share and create documents in real-time. Clients are able to use their own personal computers.
Traditional services	In-person and telephonic counselling; not digitally based services offered to EFAP clientele.
Rating of session helpfulness	Typically clients are asked at the end of each session by their counsellor to rate the session helpfulness by answering the following question:<br><br>On a scale of 0 to 10 (10 = most helpful), was this session helpful?<br><br>The counsellors can choose not to ask if they deem it clinically inappropriate.
Goal completion	Client counselling goals in the clinical file were rated as goals achieved, goals partly achieved or goals not achieved. These ratings are based on the client's reports as documented by the counsellor in the file at time of last session.
Pre/post client self-assessment	Clients are asked to rate their health and mental health at the first session and again at the last session by answering the following questions:<br><br>• In general, would you say your health is Poor/Fair/Good/Very Good/Excellent?<br><br>• In general, would you say your mental health is Poor/Fair/Good/Very Good/Excellent?<br><br>For the purposes of this study, these responses were quantified by a scale of 1-5 where 1 = Poor and 5 = Excellent.

Presenting issues	Presenting issues are identified and coded during the intake process when clients request counselling service. For this study, the 53 possible presenting issues are classified into one of four categories:<br><br>Addiction related: E.g. alcohol, smoking, drugs, etc.<br><br>Work: work performance, work relationship conflicts, violence/harassment, etc.<br><br>Individual: stress, sexuality, depression, grief etc.<br><br>Couple/family: couple relationship, child behavior, parenting, communication/conflict resolution, etc.
Client geographic location	1. Easy access: a community with existing EFAP counsellors.<br><br>2. Moderate access: a community within one hour of a centre that offers EFAP counsellors.<br><br>3. Limited/no access: a community more than one hour away from a centre that offers an EFAP counsellor.
