# Supplementary material for: Employee and Family Assistance Video Counseling Program: A Post Launch Retrospective Comparison With In-Person Counseling Outcomes
Source: Med 2 0. 2014 Apr 24;3(1):e3. doi: 10.2196/med20.3125 (PMC4084764; doi:10.2196/med20.3125)
Supplement: Supplementary file 2 [file med20_v3i1e3_app2.pdf]

Multimedia Appendix 2: Clinical intake screening process [1]

Within each request for service, there is a well-defined process to assess and understand the issues/concerns of the client and match them to a type and modality of service that best meets their needs and goals. Some considerations during the intake process include, but are not limited to:

**Clinical best practice**	Some of the services may not be appropriate for individuals in crisis, and as such they will not be offered to EFAP clientele; more appropriate clinical interventions will be identified and recommended. As well, certain types and modalities of service are preferable to address chronic, complex issues associated with serious mental health conditions.
**Age**	Some of the services noted above are not appropriate for those who are under 18, such as First Chat.
**Employment factors**	Whether a client is employed in a part-time/full-time role, retired or unemployed, working shifts, working remotely, working in various rotating locations, and/or working in a setting allowing for privacy, etc. are all variables for consideration in determining what modality of service is best suited to the client's capacity to participate in clinical programming.
**Lifestyle**	Lifestyle variables and a client's capacity to allocate time to the clinical process will be assessed at the time of intake. For example, a client who spends most of their time at the workplace with no privacy may not be best suited for telecounselling, but may be well suited for e-counselling or one of our other online modalities of service.

**Learning style**	Comfort level with the various modalities of services offered, based on prior experience, current expectation, and reported success in the past will determine the resulting recommendation(s).
