# Supplementary material for: Employee and Family Assistance Video Counseling Program: A Post Launch Retrospective Comparison With In-Person Counseling Outcomes
Source: Med 2 0. 2014 Apr 24;3(1):e3. doi: 10.2196/med20.3125 (PMC4084764; doi:10.2196/med20.3125)
Supplement: Supplementary file 3 [file med20_v3i1e3_app3.pdf]

Multimedia Appendix 3: Technological requirements for video counselling

Clients must have:

- a private email address
- high-speed internet
- web cam
- land line within reach of their computer monitor (cell phone use is allowed but clients are advised against it and are informed of the potential confidentiality risks associated with cell phone use)
- comfort with using internet and web cam, preferably experience with platforms like Skype
- a quiet private place for the appointment with no distractions or interruptions
